# Creating effective interrupted time series graphs: Review and recommendations

**DOI:** 10.1002/jrsm.1435

**Published:** 2020-07-22

**Authors:** Simon L. Turner, Amalia Karahalios, Andrew B. Forbes, Monica Taljaard, Jeremy M. Grimshaw, Elizabeth Korevaar, Allen C. Cheng, Lisa Bero, Joanne E. McKenzie

**Affiliations:** ^1^ School of Public Health and Preventive Medicine Monash University Melbourne Victoria Australia; ^2^ Clinical Epidemiology Program Ottawa Hospital Research Institute Ottawa Ontario Canada; ^3^ School of Epidemiology and Public Health University of Ottawa Ottawa Ontario Canada; ^4^ Department of Medicine University of Ottawa Ottawa Ontario Canada; ^5^ Infection Prevention and Healthcare Epidemiology Unit Alfred Health Melbourne Victoria Australia; ^6^ Faculty of Medicine and Health, School of Pharmacy and Charles Perkins Centre The University of Sydney Sydney New South Wales Australia

**Keywords:** data visualization, display of data, graph, interrupted time series, meta‐analysis, systematic review

## Abstract

**Introduction:**

Interrupted Time Series (ITS) studies may be used to assess the impact of an interruption, such as an intervention or exposure. The data from such studies are particularly amenable to visual display and, when clearly depicted, can readily show the short‐ and long‐term impact of an interruption. Further, well‐constructed graphs allow data to be extracted using digitizing software, which can facilitate their inclusion in systematic reviews and meta‐analyses.

**Aim:**

We provide recommendations for graphing ITS data, examine the properties of plots presented in ITS studies, and provide examples employing our recommendations.

**Methods and results:**

Graphing recommendations from seminal data visualization resources were adapted for use with ITS studies. The adapted recommendations cover plotting of data points, trend lines, interruptions, additional lines and general graph components. We assessed whether 217 graphs from recently published (2013‐2017) ITS studies met our recommendations and found that 130 graphs (60%) had clearly distinct data points, 100 (46%) had trend lines, and 161 (74%) had a clearly defined interruption. Accurate data extraction (requiring distinct points that align with axis tick marks and labels that allow the points to be interpreted) was possible in only 72 (33%) graphs.

**Conclusion:**

We found that many ITS graphs did not meet our recommendations and could be improved with simple changes. Our proposed recommendations aim to achieve greater standardization and improvement in the display of ITS data, and facilitate re‐use of the data in systematic reviews and meta‐analyses.

## Highlights

- Application of data visualization recommendations can improve quality of interrupted time series graphs.
- Well‐designed graphs accurately depict time series data, any impact of the interruption, and the results of the analysis.
- Well‐designed graphs facilitate data extraction for use in systematic reviews and reproducibility.
- An assessment of graphs included in interrupted time series studies (published between 2013 and 2017) found that graphs often do not meet core graphing recommendations.

## 1 INTRODUCTION

Interrupted time series (ITS) studies are a common design used in areas such as public health, health policy and health services research to examine the effects of an interruption on an outcome. The interruption could be planned, such as the roll out of a new health policy, or unplanned, such as an unintended environmental exposure. This makes ITS studies a valuable design for inclusion in systematic reviews intended to inform policy decisions. However, effective and accurate presentation of the data from ITS studies is needed to enable their inclusion in systematic reviews (including meta‐analysis) and to aid interpretation of the results from the review.

The ITS design inherently lends itself to a visual display. In an ITS study, data on a group of individuals (eg, hospital, country) are collected at multiple time points both before and after the interruption. By modeling data from the pre‐interruption period, the underlying secular trend can be established and extrapolated to the post‐interruption period, creating a counterfactual for what would have occurred in the absence of the interruption. Statistical comparisons between the counterfactual and observed data at different points post interruption can be used to estimate the short‐ and long‐term effects of the interruption. These features can be visually displayed, and in well‐designed graphs, the impacts of the interruption on the outcome will likely be evident. For these reasons, visual displays of ITS data in both primary studies, and systematic reviews of ITS studies, are a valuable part of reporting.

A further benefit of visually displaying data from an ITS study is that it allows systematic reviewers to extract the data (eg, using digitizing software) and undertake a re‐analysis. A re‐analysis may be required in the circumstance where effect estimates have been incompletely reported (ie, when an effect estimate is reported without a measure of precision^1^); where the data has been incorrectly analyzed (eg, when there has been no adjustment for autocorrelation—a common complication with time series data^2^, ^3^, ^4^); or, when the reviewer wishes to estimate the effect of the interruption using a different effect measure to that reported in the paper (eg, an estimate of level change may be reported but a reviewer may be interested in a difference in slopes or the combined effect of level and slope change). Such re‐analyses are particularly important when reviewers wish to meta‐analyze the estimated effects, where consistency in the effect measures across studies, and estimates and their standard errors from correct analyses of time series data are required.

The aim of this research is to provide recommendations for the accurate display of ITS data in primary ITS studies and systematic reviews of ITS studies. We first provide principles and recommendations for graphing ITS data (Section 2). We then examine how often graphs are used to display data in reports of ITS studies and the extent to which those graphs meet our recommendations (Section 3). Finally, we present examples of ITS graphs before and after incorporating our recommendations (Section 4).

## 2 RECOMMENDATIONS FOR GRAPHING INTERRUPTED TIME SERIES DATA

In this section we suggest recommendations along with the rationale for the visual display of results from ITS studies (Table 1). These have been informed from seminal data visualization resources, including Boers,^5^ Cleveland,^6^ Few,^7^ Lane and Sándor,^9^ Tufte^8^, ^10^, ^11^ and Yau.^12^ In forming these recommendations, we first articulated the primary purpose of an ITS graph, which we consider to be the presentation of an accurate visual depiction of the ITS data, including display of essential details for accurate extraction of the data points. Considering our purpose, the following components should be displayed:

1. the data points;
2. the interruption time;
3. pre‐interruption and post‐interruption trend lines; and
4. the counterfactual trend line.

**TABLE 1:** Recommendations for graphing interrupted time series data

Characteristic	Recommendation	Importance	References
Data Points	Plot data points. Each point should be clearly visible	Core	Boers,^5^ Cleveland,^6^ Few,^7^ Tufte^8^
	Use the same data points as were used in the analysis	Core	Lane,^9^ Tufte^10^, ^11^
	Line up data points with x‐axis tick marks	Core	Few,^7^ Tufte,^8^ Yau^12^
	Do not join data points with lines	Additional	Few,^7^ Tufte^8^
Interruption	Indicate interruption time with vertical line or shading	Core	Cleveland,^6^ Yau^12^
	Show any transition or roll‐out period with vertical lines or shading	Core	Cleveland,^6^ Lane,^9^ Tufte^11^
	Label the interruption line	Core	Tufte^8^, ^11^
Trend lines	Plot the fitted pre‐ and post‐interruption trends	Core	Cleveland,^6^ Few,^7^ Lane,^9^ Yau^12^
	Use bold and solid lines for fitted trends	Additional	Few,^7^ Yau^12^
	Match trend line with data point color	Additional	Boers,^5^ Cleveland,^6^ Few,^7^ Tufte,^10^, ^11^ Yau^12^
Counterfactual	Indicate counterfactual with a trend line	Core	Cleveland,^6^ Tufte,^10^, ^11^ Yau^12^
	Use a different line pattern for the counterfactual trend as compared with the fitted pre‐ and post‐interruption trend lines	Additional	Tufte,^11^ Yau^12^
	Match counterfactual trend line with data point color	Additional	Boers,^5^ Cleveland,^6^ Few,^7^ Tufte,^10^, ^11^ Yau^12^
Additional lines	Use a different color and symbol for each additional series	Core	Cleveland,^6^ Few,^7^ Tufte^11^
	Use neutral colors for additional lines (eg, confidence intervals)	Additional	Boers,^5^ Cleveland,^6^ Few^7^
General graph components	Show axis tick marks	Core	Tufte,^10^, ^11^ Yau^12^
	Label axes	Core	Tufte,^10^, ^11^ Yau^12^
	Align axis labels with axis tick marks	Core	Yau^12^
	Include axis titles in which the variables and units of measurement are clear	Core	Tufte,^11^ Yau^12^
	Explain all elements of the graph in the figure and/or caption	Core	Boers,^5^ Cleveland,^6^ Few,^7^ Lane,^9^ Tufte^8^, ^11^
	If used, grid lines should be faint	Additional	Boers,^5^ Few,^7^ Tufte^8^
	Use the smallest scale that incorporates all data	Additional	Boers,^5^ Cleveland,^6^ Lane,^9^ Tufte^8^
	Minimize the visual impact of additional text (eg, legends and keys)	Additional	Boers,^5^ Few,^7^ Tufte^10^, ^11^
	Use horizontal text whenever possible	Additional	Yau^12^
	Use color blind friendly colors	Additional	Boers,^5^ Tufte,^8^ Yau^12^

**FIGURE A1:**
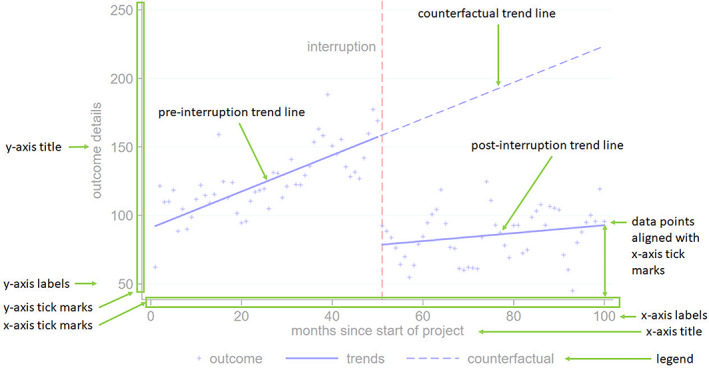
terminology used for some components of graphs [Colour figure can be viewed at wileyonlinelibrary.com]

Plotting other features of ITS analyses (eg, seasonality, confidence intervals) and features common to many graphs (eg, axis labels, additional text, legends) also need to be considered. The recommendations have been divided into those which are “core” for readers to be able to correctly interpret and extract data from the graphs; and those which are “additional,” where applying the recommendation is not required, but may aid in interpretation and enhance visual appeal of the graph. Our terminology is described in Figure A1 of Appendix. Code for creating the example graphs using Stata^13^ is available in Supporting Information S1.

### 2.1 Data points

A core recommendation for data points is to plot all the raw data points, since they form the basis of the analysis and allow examination of variation in the data along with other distributional information^5^, ^6^, ^7^, ^8^(Table 1). Further, plotting of points allows readers to extract the data, which is particularly important for systematic reviewers. The symbols chosen for plotting the data points should be clearly visible in the presence of other lines (such as trend lines) and we recommend including a center point (eg, x or +), since this facilitates accurate data extraction. Consideration should also be given to select colors from a color‐blind‐friendly palette.^5^, ^8^, ^12^

A further core recommendation is to reflect the same data as included in the analysis (eg, if data were analyzed at monthly intervals, monthly data points should be shown, rather than aggregating to a different time period for the graph).^9^, ^10^, ^11^ For data extraction to be accurate, the data points should align with the x‐axis tick‐marks, hence this is a core recommendation.^7^, ^8^, ^12^ An additional recommendation is to *not* join consecutive data points by lines. The addition of such lines can obfuscate data points, does not add relevant information, and introduces visual clutter that can obscure more important elements such as the trend lines.^7^, ^8^

### 2.2 Interruption

**FIGURE 1:**
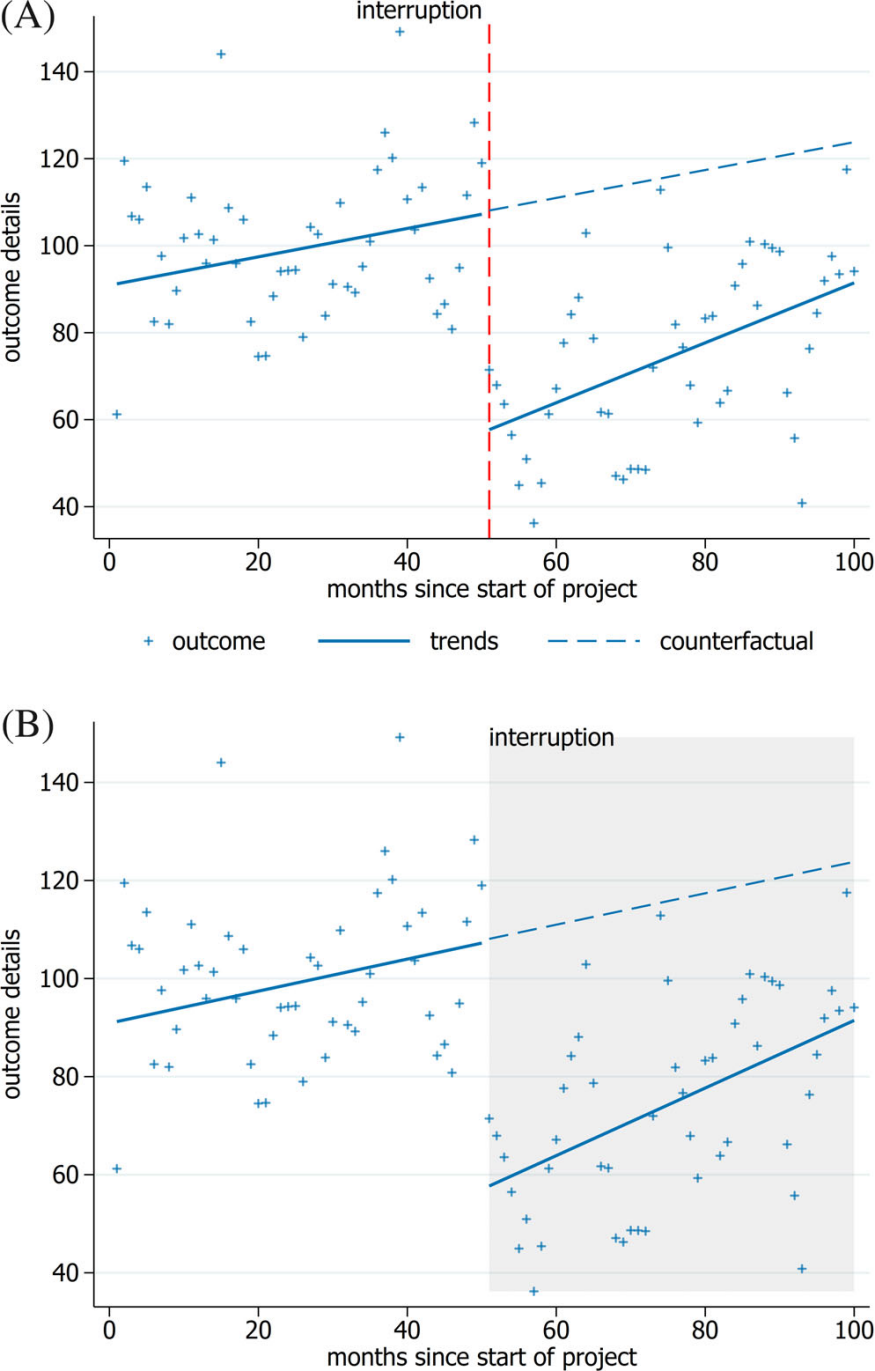
Interrupted time series graphs showing key components. The data points are plotted, and the combination of trend lines and counterfactual allow visualization of the change in level from counterfactual to post‐intervention trend as well as the change in slope. The timing of the interruption is indicated by a vertical line in, A, and a shaded area in, B. The legend is included in the first example (A) but could easily be removed to increase the size of the graph if appropriate text was used in the caption of the graph (B) [Colour figure can be viewed at wileyonlinelibrary.com]

We suggest three core recommendations for presenting the interruption (Table 1). We recommend that each interruption of the time series be shown with either a vertical line at the point of the interruption (Figure 1A) or by a light shading of a time period (Figure 1B). Plotting this line allows a reader to readily visualize the change in level at the time of the interruption.^6^, ^12^ A single line is more appropriate when the interruption is a singular event that occurs at a given time (eg, an earthquake). Shading can be useful to indicate that the interruption is taking place over the indicated time period (eg, a policy implementation) or to indicate sections that are used (or not used) in the statistical analysis (eg, a transition period). Note however, shading can add visual clutter so using light, neutral colors is recommended to avoid this.^6^, ^9^, ^11^ As interruptions are key components of an ITS design, labels should also be added to clearly indicate what each line (or area) represents, though care should be taken so that they do not obscure data points.^8^, ^11^ Further, it is important that the labels used in the graph match the terminology used in the manuscript.

### 2.3 Trend lines

**FIGURE 2:**
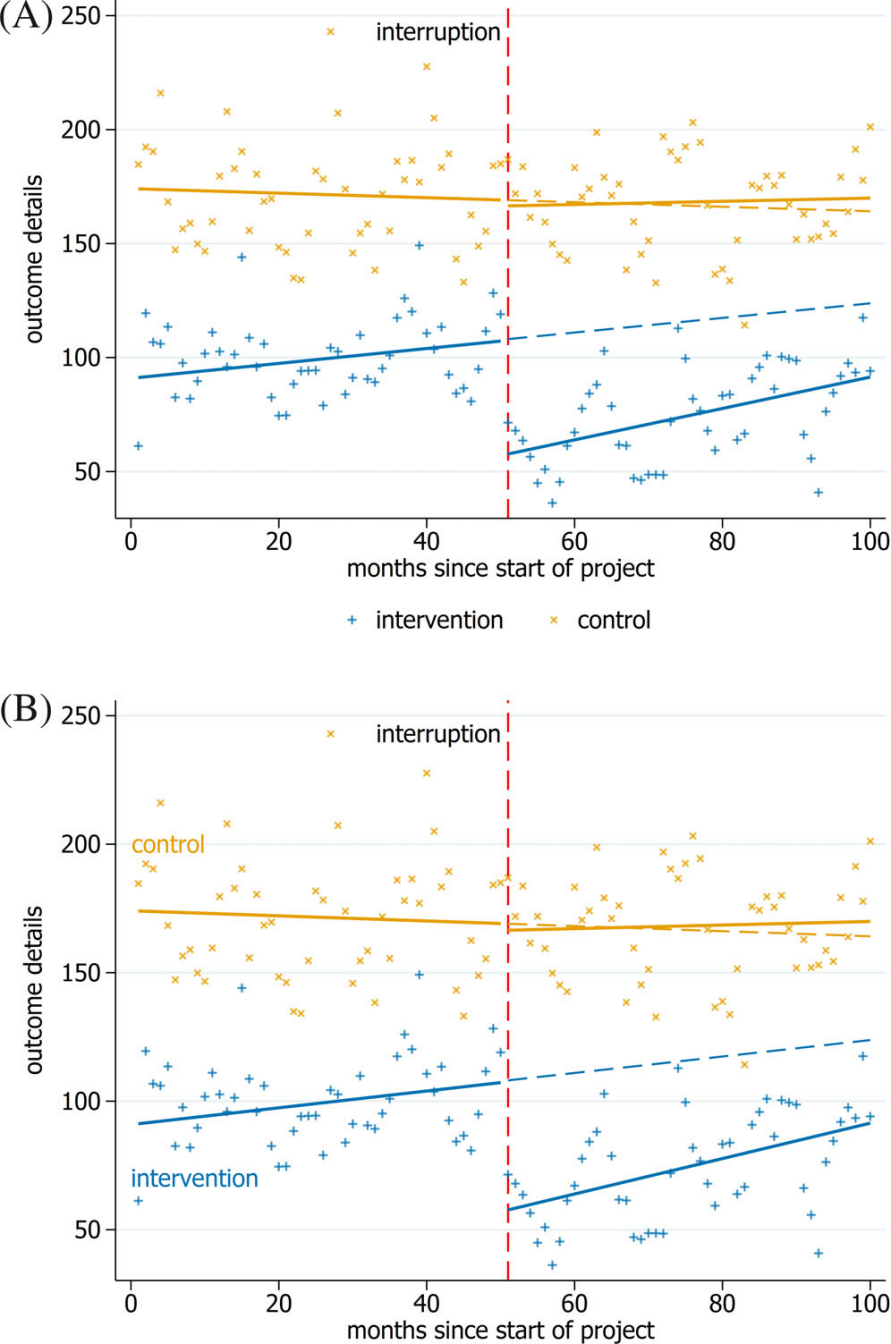
Multiple interrupted time series in a single graph. The different series can be distinguished by different colors, symbols, or ideally both, A. Care must be given to not overly clutter the graph. The legend could be omitted with extra labels on the graph, as in, B, or with text in the figure caption [Colour figure can be viewed at wileyonlinelibrary.com]

As a core recommendation, we suggest including lines to show the fitted trends, since trend lines allow the reader to readily visualize changes (or lack thereof) in the level and slope^6^, ^7^, ^9^, ^12^ (Figures 1 and 2A). Presentation of such lines allows readers to assess goodness of fit and whether an appropriate model has been fitted (eg, when straight lines are fitted but the data indicates a curvilinear line may be preferable).^6^, ^7^ Additional recommendations are to use bold and solid lines for the fitted trends, and to match the color of the pre‐ and post‐interruption trend lines with the matching data points. Use of bold and solid lines focuses attention on the trend lines^6^, ^9^, ^11^ (Figure 1). Use of the same colors for trend lines and data points visually indicates that they represent trends of the same data series, and the reader is likely to subconsciously make this association quickly.^5^, ^6^, ^7^, ^10^, ^11^, ^12^

### 2.4 Counterfactual trend line

As a core recommendation, we suggest plotting the counterfactual trend line to allow a reader to easily make a visual assessment of any change between it and the post‐interruption trend line^6^, ^10^, ^11^, ^12^ (Figure 1). The counterfactual line is formed by continuing the trend estimated from the pre‐interruption time period into the post‐interruption period. The difference in the lines indicates changes that may be due to the interruption and can be seen over the entire post‐interruption period, allowing assessment of both short‐ and long‐term effects. Additional recommendations are to plot the counterfactual trend line using the same color as the data point color, and to use a different line pattern (eg, dashed) as compared with the pre‐interruption trend. The latter makes it intuitively clear that the counterfactual is an extension of the pre‐interruption trend and is not based on observed data in that time period^5^, ^6^, ^7^, ^10^, ^11^, ^12^ (Figure 1).

### 2.5 Additional lines

**FIGURE 3:**
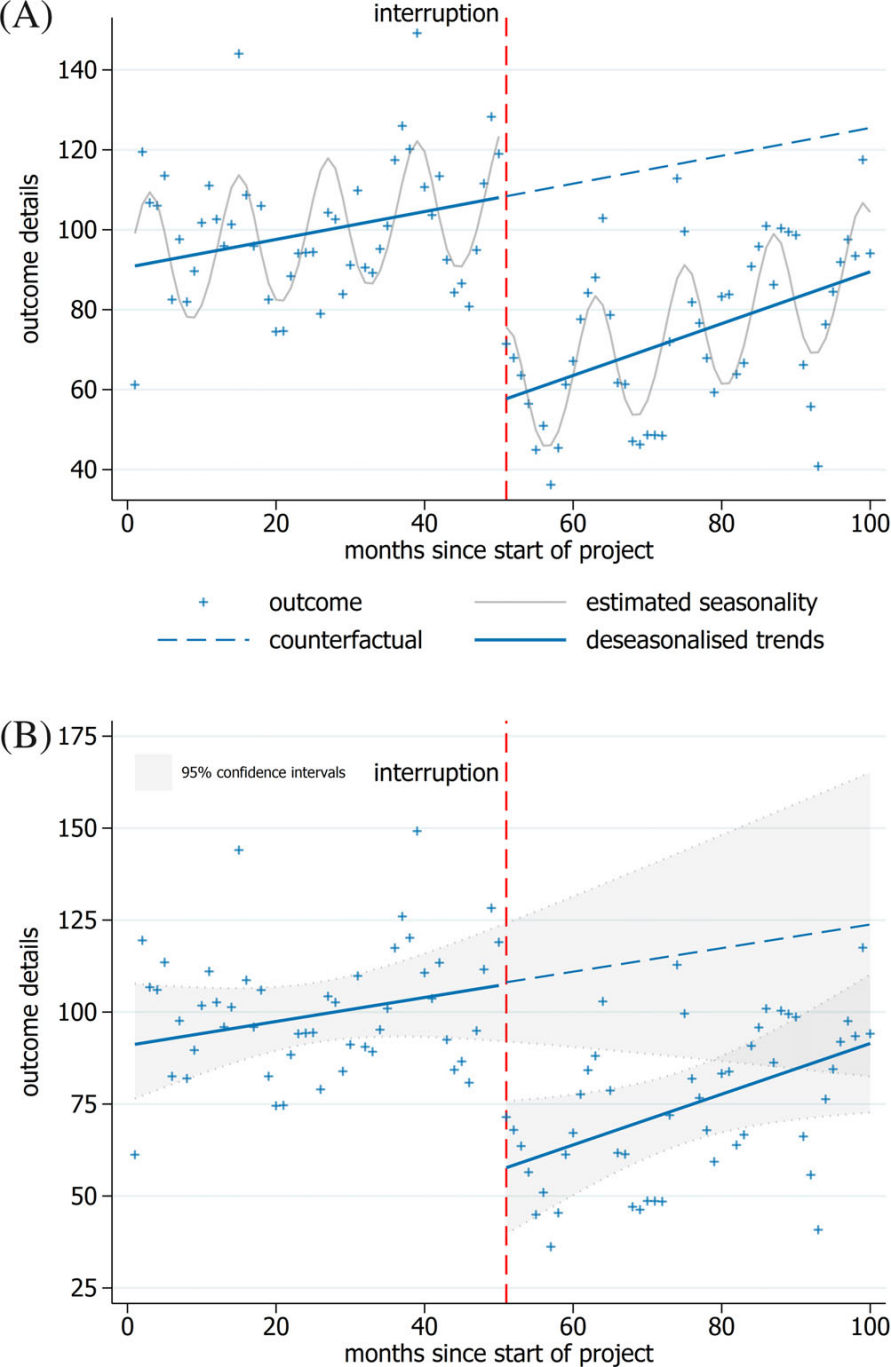
Interrupted time series graphs with additional lines. Neutral colors should be used, such as gray for the seasonality curves, A, and confidence intervals for the modeled trends, B. Note that with appropriate text in the figure caption, the legend can be removed, placing greater emphasis on the data points, B [Colour figure can be viewed at wileyonlinelibrary.com]

Additional lines may be considered in some circumstances to reflect aspects of the analysis or provide additional information. For example, in ITS studies with multiple series (eg, those with a control series), it can be helpful to plot all the series in one graph (Figure 2). Additionally, periodic trends in the data (such as seasonality) may be modeled in the analysis and these can be shown on the graph (Figure 3A). Finally, another potentially useful addition is the inclusion of confidence limits for the modeled trends (Figure 3B).

When plotting multiple series, it is important to distinguish the series. Therefore, as a core recommendation, we suggest that this be done by using a different symbol and color for each data series^6^, ^7^, ^11^(Figure 2). Symbols should be chosen so that they are easy to distinguish, this is helpful particularly when the two series overlap and for readers wishing to extract data.^6^ If multiple series are difficult to distinguish when plotted on the same graph, a side‐by‐side presentation could be useful. Plotting both graphs on the same y‐axis scale will aid comparison.

Inclusion of additional information on a graph needs to be undertaken carefully so as not to obscure the essential components of the graph or add clutter.^5^, ^6^, ^7^ For example, a seasonal trend could be added using a neutral color that does not inhibit view of the trend lines (Figure 3A). Light gray is recommended, since it does not immediately attract a reader's attention, thus allowing the more important elements to dominate.^5^, ^6^, ^7^ Similarly, confidence limits could be presented using dashed lines or with light shading in a neutral color; however, care should be taken so that shading does not obscure the data points or the interruption period shading (when used) (Figure 3B).

### 2.6 General graph components

**FIGURE 4:**
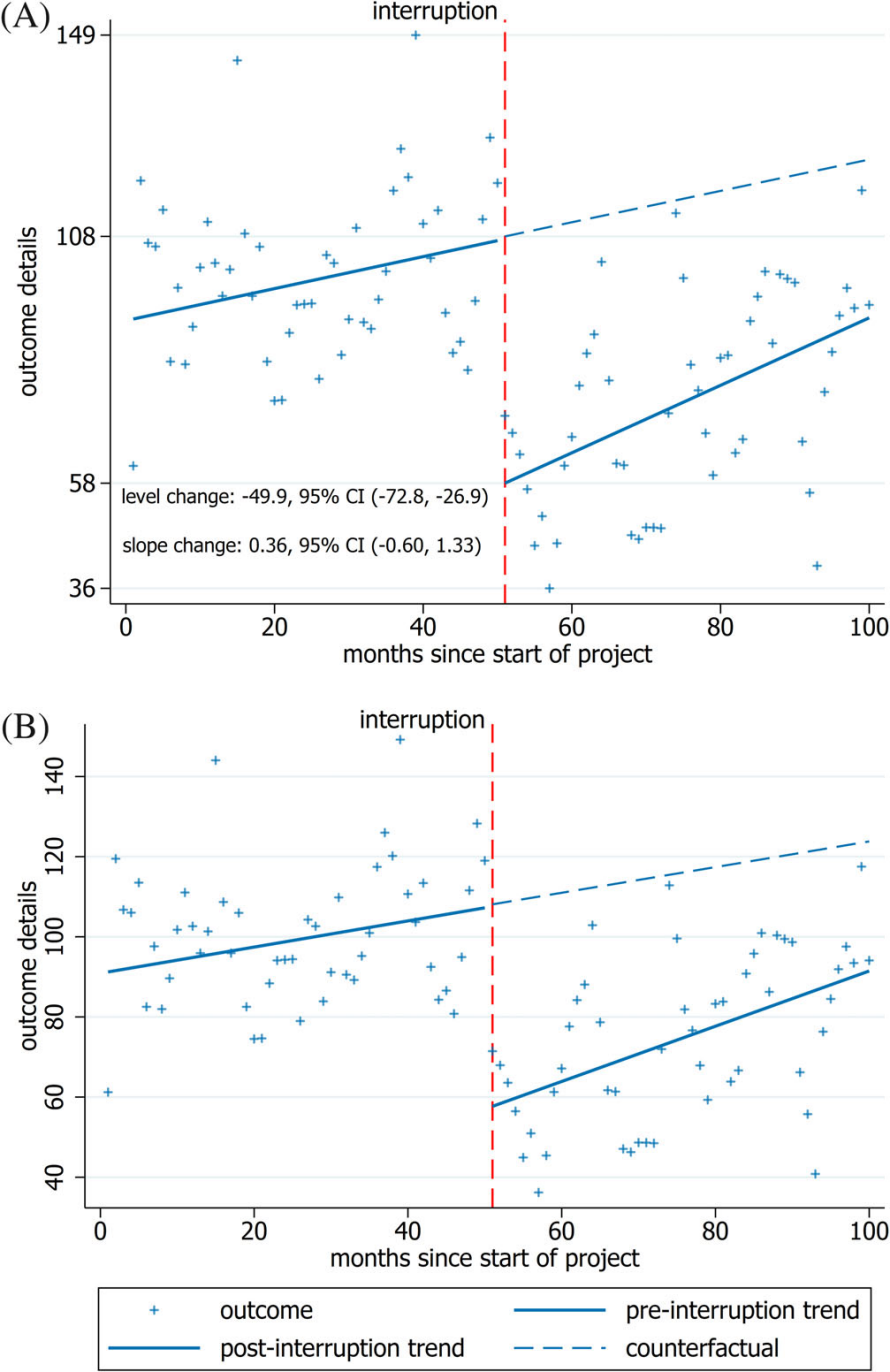
Interrupted time series graphs with additional text. By removing the legend and restricting the scale of the y‐axis to the range of data, the space available to plot the data is maximized, A. Including text showing the level and slope change with confidence intervals in (A) enables the reader to quickly access the analysis results. The horizontal grid lines and y‐axis labels in (A) have been shown at the minimum, maximum and level change points. Leaving the box around the legend in, B, unnecessarily clutters the image. Rotating the text for the y‐axis labels from vertical (B) to horizontal (A) also aids in interpretation without unduly reducing the space available to plot the data [Colour figure can be viewed at wileyonlinelibrary.com]

There are many other components of a graph (eg, axes, grid lines, legends and other additional text) that can enable the reader to more easily interpret the data. Here we focus on the components that are likely to be most helpful for aiding interpretation and those which facilitate accurate extraction of data from the graph. Core recommendations are that axis tick marks are shown and labeled, with labels aligned to the tick marks, since these are essential for accurate extraction of data points from the graph^10^, ^11^, ^12^ (Figure 4). Additionally, grid lines can be used to show elements of the data distribution or analysis (eg, minimum and maximum values, points of the level change; Figure 4A). When included, it is helpful for grid lines to be faint so that they do not detract from the rest of the graph.^5^, ^7^, ^8^

An additional recommendation is to use the smallest scale that still incorporates all of the data, since this maximizes the space for plotting the points, which adds clarity and facilitates data extraction^5^, ^6^, ^8^, ^9^ (see the comparison between Figure 4A and Figure 4B). For time series data there is no requirement to include zero on the vertical scale.^5^, ^6^, ^9^

Text can be added to graphs to provide additional information. As a core recommendation, we suggest that detailed titles should be included with the axes to ensure that the variables plotted and their units of measurement are known. Additionally, axis label text is generally easier to read if horizontal^12^ (eg, compare the horizontal axis labels in Figure 4A to the vertical axis labels in Figure 4B). Additional text in the form of a legend can be helpful but is not required when the information is included in the figure caption or on the graph itself^7^, ^9^ (eg, labeling the intervention and control series in Figure 2B).

Another example of additional text is the inclusion of effect estimates on the graph (Figure 4A). This enables readers to both visualize the level and slope change as well as quantify the values. If not included on the graph, this information could be placed in the caption ensuring that the results from the analysis are readily available.^5^, ^6^, ^7^, ^8^, ^9^, ^11^ Reporting of effect estimates should be accompanied by a measure of precision, such as a confidence interval, in order to show the uncertainty.^11^

A risk of additional text is that it can clutter the graph, obscure data points and remove attention from the plotted data. Removing extraneous components such as boxes surrounding additional text (eg, legend boxes), using a minimal number of words and using a small font can reduce this risk.^5^, ^6^, ^11^ For example, in Figure 4B the legend takes up too much space and the box around it draws attention away from the plotted data.

## 3 REVIEW OF INTERRUPTED TIME SERIES GRAPHS

### 3.1 Methods

In a previous study we undertook a review examining the design characteristics and statistical methods used in ITS studies evaluating public health interventions. Details of our methods are outlined in our protocol.^14^ In brief, we identified 200 ITS studies examining the impact of a public health intervention or exposure that has public health implications published between 2013 and 2017 and indexed on PubMed. For each ITS study, multiple outcomes were potentially eligible for inclusion. Within each outcome type category (binary, continuous, count) a prespecified hierarchy of decision rules was used to select one outcome (see Turner et al^14^ for further details). We extracted data on the study characteristics, statistical models, estimation methods, effect measures and parameter estimates, and whether a graph was included.

For the present paper, we assessed the quality of the graphs included in our review by examining whether the graphs met the core recommendations outlined in Table 1. Two reviewers undertook this assessment independently for all graphs (SLT and one of AK, EK or JEM). Frequencies and percentages of graphs meeting each of the core recommendations are tabulated.

### 3.2 Results

Our review of 200 ITS studies included 230 outcomes, for which there were 217 associated ITS graphs (Table 2). Data points were plotted in 60% (130/217) of graphs. Points were joined by lines in 64% (138/217) of the graphs, against our recommendation. Tick marks were provided on the x‐axis in most graphs (90%, 195/217). However, when tick marks were provided, the data points were aligned in only 56% (109/195) of the graphs. Joint criteria for accurate data extraction require distinct points that align with axis tick marks and labels that allow the points to be interpreted; these criteria were jointly met in only 33% (72/217) of graphs.

**TABLE 2:** Prevalence of adherence to core graphing recommendations, N = 217

Graph feature	n	%
*Data points*		
*Core*:		
Distinct individual points plotted	130	60
Tick marks on the x‐axis	195	90
Data points align with tick marks on the x‐axis (n = 195)	109	56
x‐axis labels aligned with tick marks (n = 195)	140	72
x‐axis units of measurement clear^a^	211	97
Tick marks on the y‐axis	215	99
y‐axis labels aligned with tick marks (n = 215)	215	100
y‐axis units of measurement clear^a^	148	68
Accurate data extraction possible^b^	72	33
*Additional*:		
Data points not joined by lines	79	36
*Lines*		
*Core*:		
There was a line representing the time of the interruption	158	73
There were labels for the segments or interruption line on the graph	130	60
There were lines for the trends in each segment	103	48
There was a line for the counterfactual	37	17
*Additional*:		
Multiple series were plotted	91	42
How multiple series were plotted (N = 91)		
Different color and/or pattern and different symbol	32	35
Different color and/or pattern same symbol	44	48
Same color and/or pattern different symbol	14	15
Same color and/or pattern same symbol	1	1
Other model‐based lines were plotted, for example, Seasonality curves	21	10

^a^ A graph was assessed as having a clear unit of measurement if the axis title made the unit of measurement clear, or the unit of measurement was clear from the axis labels (eg, dates).

^b^ Joint criteria for accurate data extraction requires distinct points that align with axis tick marks and labels that allow the points to be interpreted.

A distinct line was used to indicate the interruption in 73% (158/217) of the graphs. Lines were used to indicate the trends in each segment in under half of graphs (48%, 103/217), and the counterfactual was rarely plotted (17%, 37/217). The segments or interruption line were labeled in 60% of graphs (130/217). When additional series were plotted, they met our recommendation of using both a different color and symbol in 35% of the graphs (32/91). Other model‐based lines such as seasonality curves or confidence intervals were presented in 10% of the graphs (21/217).

## 4 APPLICATION OF GRAPHING RECOMMENDATIONS

In the following examples we apply our graphing recommendations to two graphs from ITS studies. These examples were identified from our review of ITS graphs (Section 3), and were selected for their potential to demonstrate many of the graphing recommendations.^15^, ^16^ Datasets were extracted from published graphs using WebPlotDigitizer which has been found to be a valid tool for data extraction.^17^, ^18^ Note that while our analytical methods may differ to those applied in the original publications, this is inconsequential for our purpose of demonstrating the impact of applying the graphing recommendations.

### 4.1 Example 1

An ITS study examined the impact of an amendment to self‐defense laws on homicide rates in Florida, United States of America.^15^ The amendments provided legal immunity to individuals that used lethal force in self‐defense situations. Four states (New York, New Jersey, Ohio, and Virginia), where the amendment to the law did not occur, were used to form a single control series. Data on the homicide rate per 100 000 people were collected from 1999 to 2015 and aggregated at monthly time intervals. A main finding of the analysis was that there was an increase in homicide rates in Florida due to the amendment.

**FIGURE 5:**
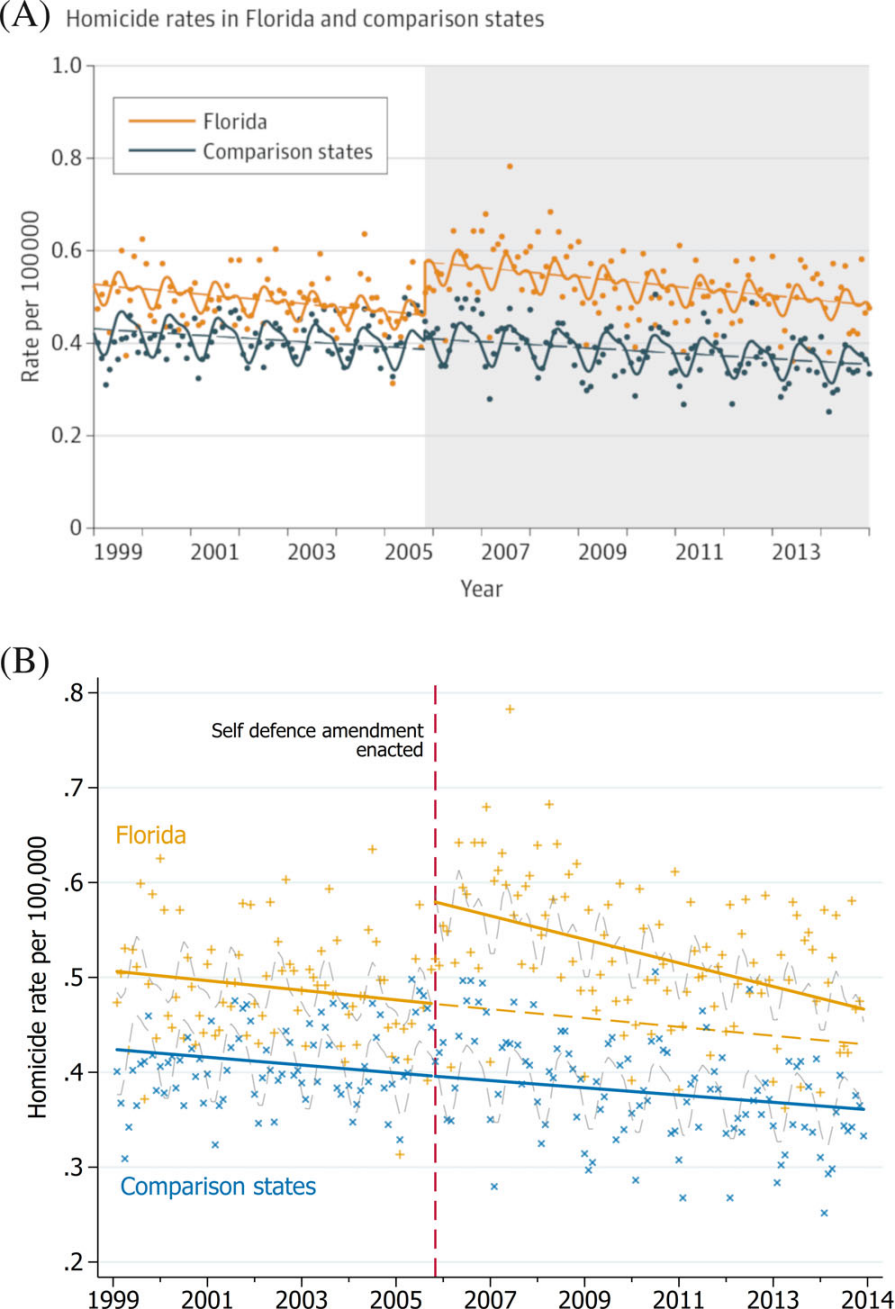
A, Published version of the graph. Reproduced with permission from JAMA Internal Medicine 2017;177(1):44‐50. doi: 10.1001/jamainternmed.2016.6811. Copyright©(2017) American Medical Association. All rights reserved. B, Revised graph using the proposed graphing recommendations [Colour figure can be viewed at wileyonlinelibrary.com]

The graph in the published paper depicts the data points, trends and seasonality for the intervention and control series (Figure 5A). The graph includes a legend to denote the series. Tick marks are included with labeled axes and the intervention time period is represented with shading.

Using our recommended guidelines would lead to a number of changes (Figure 5B). Data points have been plotted using different colors and symbols for the two series as this aids data extraction. The intervention period has been labeled and the shading has been removed to lessen the visual clutter; however, we recognize that the approach taken to identifying the interruption is one of choice and that some authors or journals may have preference for shading. Bold and solid lines have been used to prominently display the trends in the pre and post interruption periods. The counterfactual lines have been added as dashed lines to allow visual assessment of the level and slope changes over time. Seasonal effects are still shown, though they are now plotted in light gray dashed lines so as not to obscure the more important data points and trend lines. The x‐axis has been adjusted so that the points are more clearly aligned with the tick marks to facilitate data extraction. To free up more space in the graph, the x‐axis title has been removed, since it is clear from the x‐axis label what the unit of measurement is (ie, years). For a similar reason, the range of the y‐axis has been changed from 0 to 1 to 0.2 to 0.8. An increase in the amount of space available for the data will, again, facilitate data extraction. The word “homicide” has been included in the y‐axis title to provide a more detailed description of the outcome. Finally, the legend has been removed, but information from the legend has been incorporated into the graph by labeling the series with matching colored text.

### 4.2 Example 2

An ITS study examined the impact of a physician call system change on hospital readmissions in Sunnybrook Health Sciences Centre in Toronto, Canada.^16^ The intervention involved changing how hospital admissions were distributed to inpatient physician teams. Before the system change admissions were concentrated to a single team during a given 24‐hour period, after the change, admissions were distributed over all teams each day. Data on the proportion of 4‐week readmissions were collected from 2004 to 2013 and aggregated to monthly time intervals. A main finding of the analysis showed that there was an increase in the proportion of readmissions following the call system change.

**FIGURE 6:**
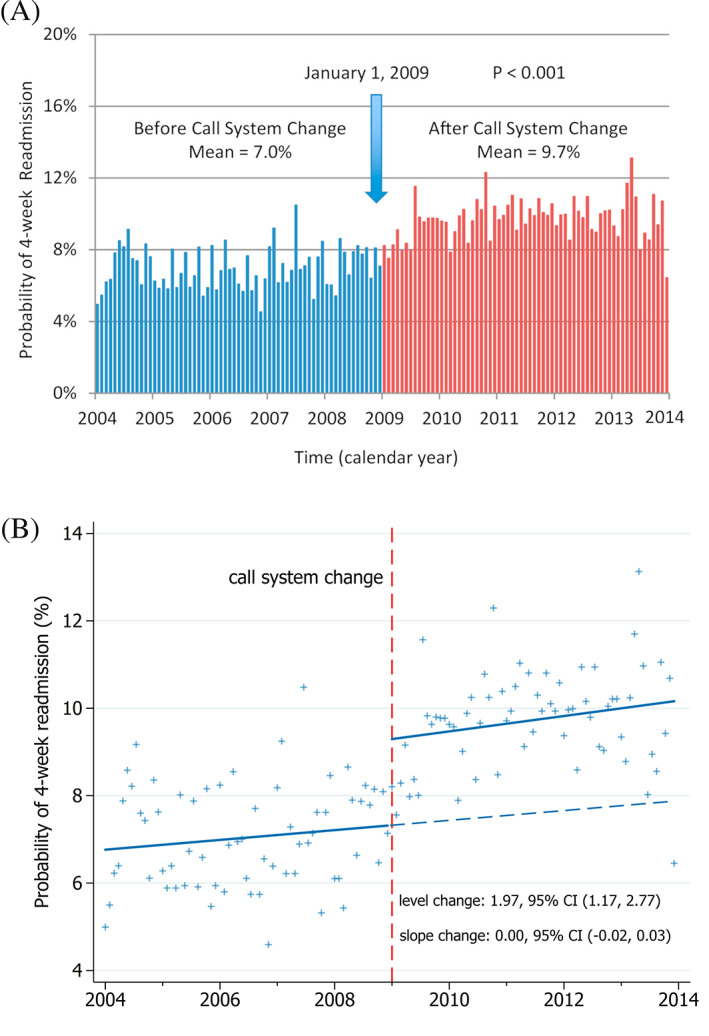
A, Published version of the graph. Reprinted from The American Journal of Medicine, 2016;129(7):706‐14.e2, Hospital Readmissions Following Physician Call System Change: A Comparison of Concentrated and Distributed Schedules, Copyright©(2016) with permission from Elsevier. B, Revised graph using the proposed graphing recommendations [Colour figure can be viewed at wileyonlinelibrary.com]

The graph in the published paper uses vertical bars to indicate the monthly proportions (Figure 6A). Colored bars are used to represent the period before (blue) and after (red) the call system change. Text is used to denote the time of the call system change as well as the mean values of the monthly proportions in each segment (pre‐ and post‐interruption) and a *P*‐value for the difference in these mean pre‐ and post‐interruption proportions. Tick marks are included with labeled axes.

Using our recommended guidelines would lead to a number of changes (Figure 6B). The data points have been plotted, which allows the spread of the data to be more easily seen, allows the data to be extracted and reduces the visual clutter. The interruption has been represented by a vertical line which is labeled. The counterfactual and trend lines have been plotted, allowing the reader to more easily see the impact of the intervention. The x‐axis has been adjusted so that the points are more clearly aligned with the tick marks to facilitate data extraction. The range of the y‐axis has been decreased to allow the data to fill the available space. Using additional text, the level and slope changes are given, along with 95% confidence intervals.

## 5 DISCUSSION

In this study we have proposed a set of recommendations for graphing ITS data. Using a sample of graphs identified from a review of ITS studies,^1^ we assessed whether the graphs met our recommended reporting items. We found that, in general, ITS graphs did not meet our core recommendations and could be improved. We demonstrated the impact of applying the recommendations to two examples.

These recommendations are of relevance to authors of ITS studies and systematic reviewers. Improvement in visual representation of ITS data in primary studies will not only facilitate better understanding of the data for readers but will allow a greater number of ITS studies to contribute to meta‐analyses. Further, even in systematic reviews where meta‐analysis of ITS data may not be possible, if data can be extracted from the graphs in the primary studies, and re‐graphed in a standardized format (through use of our recommendations) in the systematic review, this may aid interpretation. Re‐graphing the data in a standardized way overcomes the many varied visual displays of ITS data that are found in the literature.

Our recommendations were underpinned by a twofold purpose: (a) to provide an accurate visual description of the ITS data and, (b) to display essential details for accurate extraction of the data points. For many study designs, the connection between these purposes may not seem apparent. For example, in most randomized trials (except perhaps for small crossover trials), the individual observations are not plotted, and nor would it be a sensible suggestion to extract data from such graphs. In the case of ITS studies, however, most publications include a graph,^1^ the series typically do not include a large number of data points to extract,^1^ and extraction enables re‐analysis of the data that may overcome some of the shortcomings that are commonly observed in the analysis of ITS data (eg, reporting of incomplete effect estimates, no adjustment for autocorrelation^1^, ^2^, ^19^, ^20^). Further, while the purposes differ, they lead to consistent recommendations. For example, the recommendation to plot data points, while necessary for data extraction, is also a recommendation for good data visualisation.^5^, ^6^, ^7^, ^8^

General recommendations for good graphing proposed in the visual display literature informed our recommendations (Boers,^5^ Cleveland,^6^ Few,^7^ Lane,^9^ Tufte^8^, ^10^, ^11^ and Yau^12^). We considered how these recommendations should be modified for graphing data from ITS studies. While our approach to developing the recommendations was grounded in the visual display literature, other approaches (such as consensus‐based methods) could equally have been adopted, and may have resulted in different recommendations or a different emphasis on which items were recommended as core or additional. Future research that examines readers' understanding of alternative displays of the graph components will be helpful for refining the recommendations (eg, whether the use of connecting lines between points facilitates visual detection of autocorrelation).

Finally, our recommendations have been developed primarily considering the graphing of ITS data where segmented linear regression models are fitted. While it is likely that most recommendations will apply when other models and statistical methods are adopted (eg, plot data points, indicate interruption time), some recommendations may not apply or need to be adapted. For example, if a forecasting method is used,^21^ a post‐interruption trend may not be estimated, and therefore the recommendation to plot this trend line does not apply.

## 6 CONCLUSION

Graphs are useful for visually displaying the data and results from ITS studies. Well‐designed graphs allow accurate data extraction, and therefore re‐use of the data in systematic reviews and meta‐analyses. The proposed set of recommendations for graphing ITS studies aims to achieve greater standardization and improvement in the visual presentation of ITS data.

## CONFLICT OF INTEREST

The author reported no conflict of interest.

## AUTHOR CONTRIBUTIONS

Simon L. Turner conceived the study, reviewed data visualization resources, proposed the first set of recommendations and generated the computer code. Amalia Karahalios, Elizabeth Korevaar, Joanne E. McKenzie and Simon L. Turner extracted the data used in the review. Simon L. Turner wrote the first draft of the manuscript, with contributions from Joanne E. McKenzie. Simon L. Turner, Amalia Karahalios, Andrew B. Forbes, Elizabeth Korevaar, Monica Taljaard, Jeremy M. Grimshaw, Allen C. Cheng, Lisa Bero and Joanne E. McKenzie refined the recommendations, contributed to revisions of the manuscript and take public responsibility for its content.

## Supporting information


**Appendix S1** Supporting Information.

## Data Availability

Data sharing is not applicable to this article as no new data were created or analyzed in this study.
